# Recent natural selection conferred protection against schizophrenia by non-antagonistic pleiotropy

**DOI:** 10.1038/s41598-023-42578-0

**Published:** 2023-09-19

**Authors:** Javier González-Peñas, Lucía de Hoyos, Covadonga M. Díaz-Caneja, Álvaro Andreu-Bernabeu, Carol Stella, Xaquín Gurriarán, Lourdes Fañanás, Julio Bobes, Ana González-Pinto, Benedicto Crespo-Facorro, Lourdes Martorell, Elisabet Vilella, Gerard Muntané, María Dolores Molto, Jose Carlos Gonzalez-Piqueras, Mara Parellada, Celso Arango, Javier Costas

**Affiliations:** 1 Department of Child and Adolescent Psychiatry, Institute of Psychiatry and Mental Health, Hospital General Universitario Gregorio Marañón, Calle Ibiza, 43, 28009 Madrid, Spain; 2 Instituto de Investigación Sanitaria Gregorio Marañón (IiSGM), Madrid, Spain; 3 CIBERSAM, Centro Investigación Biomédica en Red Salud Mental, Madrid, Spain; 4 Language and Genetics Department, Max Planck Institute for Psycholinguistics, Nijmegen, The Netherlands; 5 School of Medicine, Universidad Complutense, Madrid, Spain; 6 Department of Evolutionary Biology, Ecology and Environmental Sciences, Faculty of Biology, University of Barcelona, Barcelona, Spain; 7 Faculty of Medicine and Health Sciences - Psychiatry, Universidad de Oviedo, ISPA, INEUROPA, Oviedo, Spain; 8 BIOARABA Health Research Institute, OSI Araba, University Hospital, University of the Basque Country, Vitoria, Spain; 9 Department of Psychiatry, Hospital Universitario Virgen del Rocío, Universidad de Sevilla, Seville, Spain; 10 Hospital Universitari Institut Pere Mata, IISPV, Universitat Rovira I Virgili, Reus, Spain; 11 Department of Genetics, University of Valencia, Campus of Burjassot, Valencia, Spain; 12 Department of Medicine, Universitat de València, Valencia, Spain; 13 Fundación Investigación Hospital Clínico de Valencia, INCLIVA, 46010 Valencia, Spain; 14 Instituto de Investigación Sanitaria (IDIS) de Santiago de Compostela, Complexo Hospitalario Universitario de Santiago de Compostela (CHUS), Servizo Galego de Saúde (SERGAS), Santiago de Compostela, Galicia, Spain

**Keywords:** Genome evolution, Genome-wide association studies, Behavioural genetics, Evolutionary genetics

## Abstract

Schizophrenia is a debilitating psychiatric disorder associated with a reduced fertility and decreased life expectancy, yet common predisposing variation substantially contributes to the onset of the disorder, which poses an evolutionary paradox. Previous research has suggested balanced selection, a mechanism by which schizophrenia risk alleles could also provide advantages under certain environments, as a reliable explanation. However, recent studies have shown strong evidence against a positive selection of predisposing loci. Furthermore, evolutionary pressures on schizophrenia risk alleles could have changed throughout human history as new environments emerged. Here in this study, we used 1000 Genomes Project data to explore the relationship between schizophrenia predisposing loci and recent natural selection (RNS) signatures after the human diaspora out of Africa around 100,000 years ago on a genome-wide scale. We found evidence for significant enrichment of RNS markers in derived alleles arisen during human evolution conferring protection to schizophrenia. Moreover, both partitioned heritability and gene set enrichment analyses of mapped genes from schizophrenia predisposing loci subject to RNS revealed a lower involvement in brain and neuronal related functions compared to those not subject to RNS. Taken together, our results suggest non-antagonistic pleiotropy as a likely mechanism behind RNS that could explain the persistence of schizophrenia common predisposing variation in human populations due to its association to other non-psychiatric phenotypes.

## Introduction

Schizophrenia is a complex mental disorder characterized by psychosis (i.e. delusions or hallucinations), social and emotional withdrawal, and cognitive deficits. Although the typical onset does not occur until adolescence or early adulthood, epidemiological and molecular evidence has consistently described the early neurodevelopmental nature of the disorder^1–3^. Affecting around 0.5% of the human population^4^, schizophrenia is associated with increased mortality^5^ and a low rate of recovery of only 13.5%^6^. Moreover, schizophrenia has been associated with high rates of comorbid illnesses such as coronary heart disease, stroke, diabetes, respiratory diseases^5,7^ and a 15% rate of unnatural deaths, including suicide^5^.

Family, twin, and adoption studies have estimated schizophrenia heritability to be 65–80%^8^, thus corroborating the major contribution of genetic variability to the development of the disorder. Although both rare copy number variants (CNVs)^9^ and disruptive mutations^10–12^ importantly contribute to the polygenic architecture of schizophrenia, genome-wide association studies (GWAS) have demonstrated that contribution from common genetic variation (mainly single nucleotide polymorphisms, SNPs) account for up to a half of the genetic variance in liability^12,13^. Methods capturing the cumulative risk such as polygenic risk scores (PGS)^14^ or linkage disequilibrium (LD) score regression (LDSC)^15^ have demonstrated an uneven distribution of schizophrenia genetic heritability across the genome, with enrichment of synaptic or brain-related pathways^16–18^, even though predisposing *loci* are genome-wide distributed.

Given the reduced fertility^19^ and the early mortality associated with schizophrenia^5^, negative selection should result in the purge of deleterious alleles that contribute to the disorder. However, the persistence of common predisposing alleles and comparable prevalence rates of schizophrenia in human populations suggests an evolutionary paradox^20,21^. Some authors have attempted to explain this paradox by balanced selection, by which schizophrenia risk alleles could also provide advantages under certain environments^22^. In this sense, a recent study described that schizophrenia alleles are linked with creativity in the general population^23^, thus supporting classical theories that proposed schizophrenia vulnerability as a price to pay for the development of language and abstract or creative thinking^24,25^. Recent works, however, have shown strong evidence against a positive selection of predisposing *loci*^26–28^.

Most of the previous studies addressing the evolutionary nature of schizophrenia lack a clear dynamic perspective of the environment during human evolution. Classical theories stating schizophrenia as a by-product of human ancestral evolution may not be relevant to explain the recent evolution of schizophrenia predisposing alleles^24,25^. In this context, the study of human populations offers a unique opportunity to address recent natural selection (RNS)^29,30^ that has taken place since African populations initiated dispersion into Eurasia around 60,000–100,000 years ago^31–33^. A recent study has demonstrated that genetic predisposing variants to schizophrenia exert similar risks across East Asian and European populations, thus pointing to a shared genetic basis for schizophrenia regardless of the ethnic or cultural backgrounds^34^. However, at the same time, SNPs associated with schizophrenia display greater population differentiation than matched control SNPs^29^, and some are reportedly even population-specific^35,36^. This scenario suggests that recent population differentiation events could have shaped the allelic distribution of schizophrenia polygenic variation across populations.

Moreover, given the high comorbidity rates of schizophrenia with other mental and somatic conditions and the pleiotropic effects of its predisposing *loci*^37,38^, selective forces for other traits could have acted to differentiate human populations across schizophrenia predisposing alleles, thus generating uneven schizophrenia genetic risks across populations. For instance, the risk allele of *SLC39A8*, a genome-wide significant gene in schizophrenia^39^, could have arisen after the human expansion to Europe to ease adaptation to colder climates by reducing blood pressure and risk of hypertension^22,40^. Older environments, however, could have promoted different genetic adaptations. In this sense, the schizophrenia alleles that have emerged and rose their frequency due to polygenic adaptation after human divergence from Neanderthal (500,000–700,000 years ago)^41,42^ may not be adaptative throughout modern environments, and therefore subject to selective pressures in modern humans.

The classical approach to discover RNS signals has consisted of seeking variants with large allele frequency differences between populations^29^. Many of the available methods are based on *F*_*ST*_ statistical tests, which need an a priori classification of studied individuals in populations^43^. However, many methods do not account for the hierarchical population structure due to the unequal differentiation within populations, which may lead to detection of many false positives^44^. Moreover, classification in fixed population groups may be challenging when population ascertainment does not reveal differentiated clusters or individuals are misclassified^45,46^. Some individual-based methods performing genome-wide selection sweeps based on principal component analysis (PCA) have been recently developed to address these challenges^47,48^.

**Figure 1:**
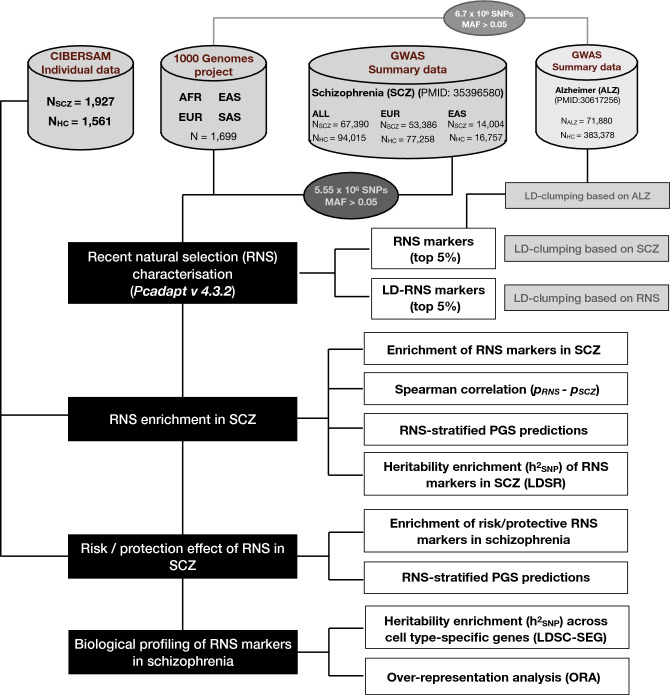
Workflow of the analytic pipeline. We used sequencing data from 1000 Genomes Project phase 3 outbred and unrelated subjects (N = 1699 European (EUR), East Asian (EAS), Southern Asian (SAS) and African (AFR) individuals; American individuals were not used due to admixed ancestry) and GWAS summary statistics from schizophrenia^49^. ancestry-specific summary statistics for European and East Asian subjects were used for additional sensitivity analyses. Overlapping variants from both sources (5,554,437 SNPs) were included in the subsequent analyses. Throughout the whole process, markers of RNS were considered as the top 5% LD-independent variants with the highest probability of being markers of natural selection (RNS markers; N_SNPs_ = 8679; LD-clumping based on SCZ). As a sensitivity analysis, we used top 5% LD-independent variants as RNS markers after LD-clumping based on RNS (LD-RNS markers; N_SNPs_ = 6262). Enrichment of RNS was also tested in Alzheimer’s disease as a methodological control. Enrichment, effect-stratified analysis (bias towards risk or protection), and biological profiling of RNS in schizophrenia were performed as described in Methods. RNS-stratified polygenic score analyses (PGS_SCZ_) were performed in an independent Spanish case–control sample (CIBERSAM; N_SCZ_ = 1927; N_HC_ = 1561) and previous SCZ GWAS^39^ as discovery sample. LDSC-SEG = LDSC applied to specifically expressed genes.

Here we aimed to perform a comprehensive approach to evaluate the effect of RNS on common genetic variation predisposing to schizophrenia (Fig. 1). First, by using *pcadapt*^48^, a recently developed PCA-based method, we described a significant association between RNS signatures and schizophrenia. Second, by focusing on the derived alleles arisen during human evolution, we demonstrated a significant trend toward fixation of derived protective alleles. Third, we explored the biological and cell-type enrichments of the RNS markers associated with schizophrenia and observed less brain and neuronal specificity than across markers not subject to RNS. Finally, among the derived alleles arisen during recent evolution, we observed greater pleiotropy for protective than for risk alleles, thus suggesting that recent protective selection to schizophrenia is related to other phenotypes resulting from recent human adaptation to different environments.

## Results

### RNS signatures across the genome

We assessed the signatures of recent polygenic adaptation across the genome after the human diaspora out of Africa (about 100 kya^31^). *Pcadapt*^48^ was used to study RNS signals across human populations from 1000 Genomes Project data. Up to 3 principal components (PCs) were kept based on the proportion of variance explained (Supplementary Fig. 1). The first PC separates African populations from the rest, while the second and third PCs differentiate between Asian and European populations (Supplementary Fig. 2), similar to previous results described using 1000 Genomes phase 1 data^47^.

RNS was characterized across the genome (Supplementary Fig. 3) and RNS *p*-values for each variant were computed (*p*_*RNS*_). A marked accumulation of SNPs subject to RNS (*p*_*RNS*_ < 0.05) was detected (Supplementary Fig. 4–5, one-sample Kolmogorov–Smirnov test *p <* 2 × 10^−16^).

In addition, to rule out the possibility that many of the described RNS markers were false positives due to discontinuous PC space^50^, we estimated *Fst* statistical parameters to describe adaptation selection markers between African and European populations (Supplementary Data 6). A great consistency was observed between *Fst* and *Pcadapt* selection signals: 98% and 73% of the top 100 selection markers estimated by *Fst* overlapped with the top 5% and 1% of *Pcadapt* RNS markers described (Supplementary Data 6).

### Enrichment of selection signatures across schizophrenia associated *loci*

We evaluated the relationship between RNS and schizophrenia predisposing variation from the latest schizophrenia GWAS^49^ using a comprehensive set of analyses (Fig. 1).

**Figure 2:**
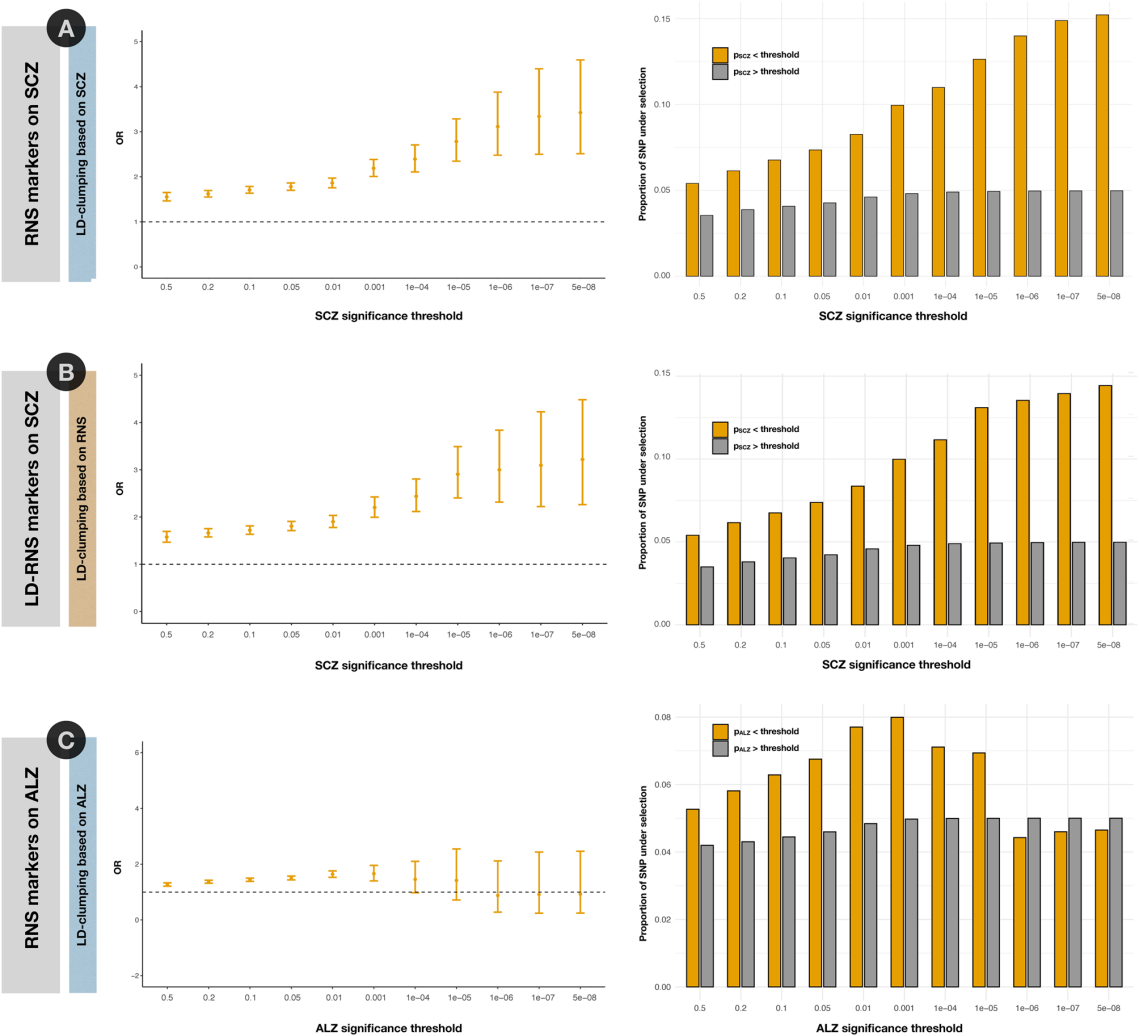
Enrichments for RNS signals in schizophrenia GWAS. Fisher’s exact tests were performed to study the enrichment of the RNS markers (LD-clumping based on *p*_*SCZ*_; top 5% of most selected SNPs; N_SNPs_ = 8679) and LD-markers (LD-clumping based on *p*_*RNS*_; top 5% of most selected SNPs; N_SNPs_ = 6262) across schizophrenia GWAS thresholds^49^. (**A**) OR (95%CI) for the enrichment and proportion of 8679 RNS markers across the different schizophrenia (SCZ) GWAS thresholds. (**B**) OR (95%CI) for the enrichment and proportion of 6262 LD-markers markers across the different schizophrenia GWAS thresholds. (**C**) OR (95%CI) for the enrichment and proportion of RNS markers across the different Alzheimer’s disease (ALZ)^51^ GWAS thresholds. LD-independent SNPs were considered after clumping based on ALZ *p*-values. See Supplementary Data 3 for detailed results.

First, we analyzed the enrichment of selection signals across schizophrenia GWAS significance thresholds. To perform well-powered analyses, we considered the 5% of LD-independent SNPs with the lowest *p*_*RNS*_ as RNS markers (N_SNPs_ = 173,701; LD-clumping based on p_SCZ_, Supplementary Data 1) and compared them with the remaining LD-independent SNPs. We observed an increasingly significant enrichment for RNS markers (N_SNPs_ = 8679) across the most stringent schizophrenia GWAS thresholds (p_SCZ_ threshold < 5 × 10^–8^, Fisher’s exact test *p =* 4.40 × 10^–13^; OR (CI 95%) = 3.45 (2.51, 4.59); Fig. 2A; Supplementary Data 3). To ensure that these associations were not artefactual due to the LD-clumping based on SCZ *p*-values, we selected the 5% of LD-independent SNPs with the lowest *p*_*RNS*_ after LD-clumping based on RNS *p*-values (LD-RNS markers; *N*_*SNPs*_ = 6262; Supplementary Data 1). Again, schizophrenia genome-wide significant (GWS) *loci* have a notably increased probability to overlap with one of the 6262 LD-RNS markers described (Fisher’s exact test *p =* 8.38 × 10^−10^; OR (CI 95%) = 3.22 (2.26, 4.48); Fig. 2B; Supplementary Data 3). Moreover, to ensure no methodology bias is present, enrichment analyses were repeated using GWAS data from Alzheimer’s disease^51^, a related brain disorder^52^ that may escape natural selection due to its late onset. LD-clumping was performed based on Alzheimer’s GWAS *p*-values. No significant enrichment between RNS and Alzheimer’s disease was observed (Supplementary Data 3; Fig. 2C) using the same procedure described above for schizophrenia.

We then assessed the partial correlation between schizophrenia and RNS across the set of LD-independent SNPs nominally associated with SCZ, while controlling for their minor allele frequency on the 1000 Genomes Project data (*p*_*SCZ*_ < 0.05; N_SNPs_ = 41,079; LD-clumping based on *p*_*SCZ*_) and found a significant correlation (Spearman-rho = 0.034; *p =* 7.03 × 10^–12^). This result was confirmed by conducting 10,000 random permutations of the GWAS results with respect to the *p*_*RNS*_ values and testing whether the observed correlation was significantly higher than the null distribution (*p*_*perm*_ = 1.3 × 10^–3^; Supplementary Data 4). A similar correlation pattern between schizophrenia and RNS was observed after LD-clumping based on RNS (Spearman-rho = 0.075; *p =* 5.12 × 10^–13^). We repeated this analysis using GWAS data from Alzheimer’s disease and observed no significant correlation with RNS (Supplementary Data 4).

Heritability enrichment analyses using LDSC showed a significant SNP-based heritability enrichment (*h*^2^_*SNP*_) of schizophrenia across the RNS markers (*h*^*2*^_*SNP*_ enrichment (CI 95%) = 1.31 (1.13 – 1.50); *p =* 1.19 × 10^–3^; Supplementary Data 5).

**Figure 3:**
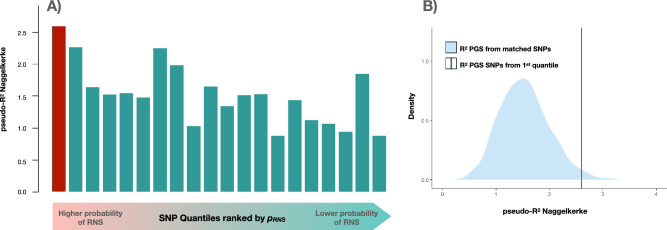
Schizophrenia Polygenic score predictions (PGS_SCZ_) in an independent cohort stratified by the probability of the SNPs to be subject to RNS. (**A**) PGS_SCZ_ predictions (% pseudo-R^2^) on an independent schizophrenia case–control sample (CIBERSAM; N_SCZ_ = 1927; N_HC_ = 1561) using 20 subsets of SNPs ranked by their probability to be subject to RNS (*p*_*RNS*_) are displayed. For this analysis, we used the most significant schizophrenia threshold in the PGS_SCZ_ analysis using whole genome data (*p*_*SCZ*_ < 0.2; N_SNPs_ = 61,040). LD-clumping based on p_SCZ_ (r^2^ < 0.1; 500 kb) was performed for PGS estimations. (**B**) PGS_SCZ_ performance (% pseudo-R^2^) using variants from the first RNS quantile (SNPs with the highest probability to be subject to RNS; *N*_*SNPs*_ = 3052) was compared against the distribution of PGS_SCZ_ Naggelkerke's pseudo-R^2^ obtained by selecting the same number of matched SNPs from the remaining 95%. A total of 1000 sets of matched SNPs were randomly drawn from SNPs matched to the variants from the first RNS quantile based on MAF, number of SNPs in LD, distance to nearest gene, and gene density. See Supplementary Data 6 for detailed results.

We then assessed schizophrenia polygenic score prediction (PGS_SCZ_) in an independent case–control cohort (CIBERSAM; N_SCZ_ = 1927; N_HC_ = 1561; Supplementary Data 6). For this analysis, we used the PGS_SCZ_ threshold with the most significant association with schizophrenia in the analysis using whole genome data (*p*_*SCZ*_ < 0.2; N_SNPs_ = 61,040). After dividing the resultant schizophrenia predisposing variation into 20-quantiles (N_SNPs_ = 3052) ranked by *p*_*RNS*_, we observed a reduction of the explained variance in the case–control cohort when moving to higher *p*_*RNS*_ (linear regression t = − 3.78; *p =* 0.0013; Fig. 3A). To account for the likely bias of the genomic properties of selection markers, we also compared PGS_SCZ_ using variants from the first quantile against PGS_SCZ_ using 1000 sets including the same number of matched SNPs selected from the rest of variants. Matched SNPs were selected accounting for MAF, number of SNPs in LD (LD buddies), distance to nearest gene, and gene density (see Methods). PGS_SCZ_ prediction from the first quantile was found to be significantly higher than the distribution of 1000 predictions from matched SNP selections (*p =* 0.019; Fig. 3B).

Moreover, to ensure our PGS_SCZ_ predictions using variants subject to RNS were not biased by the presence of Long-Range LD regions^53^, we repeated the PGS_SCZ_ analysis after excluding Long-Range LD regions previously described^53^ (*p*_*SCZ*_ < 0.2; N_SNPs_ = 60,407). Again, a similar reduction of the explained variance towards higher *p*_*RNS*_ was found (t = − 4.64; *p =* 2 × 10^–3^). Also, PGS_SCZ_ prediction from the first quantile was found to be significantly higher than the distribution of 1000 predictions from the remaining SNPs (*p =* 0.033; Supplementary Data 6).

### Enrichment of schizophrenia predisposing variation is also overrepresented in RNS markers within European populations

Given the above-described relationship between schizophrenia and RNS markers across human populations, we aimed to study whether RNS markers related with local adaptation within European or East Asian populations were also enriched in schizophrenia predisposing *loci* across these populations.

Following a similar methodology, we considered the 5% of LD-independent SNPs with the lowest *p*_*RNS*_ as RNS markers within European (N_SNPs_ = 139,668; N_samples_ = 130,644; LD-clumping based on p_SCZ;_ Supplementary Data 1C) or East Asian (N_SNPs_ = 179,669; N_samples_ = 30,761; LD-clumping based on p_SCZ;_ Supplementary Data 1D) populations and compared them with the remaining LD-independent SNPs. We observed an increasingly significant enrichment for European-based RNS markers (N_SNPs_ = 6983) across the most stringent schizophrenia GWAS thresholds (p_SCZ_ threshold < 5 × 10^–8^, Fisher’s exact test *p =* 2.30 × 10^–6^; OR (CI 95%) = 2.76 (1,83, 4.04); Supplementary Data 3). In case of the less powered test based on East Asian populations, there is also a trend for enrichment of schizophrenia *loci* across East Asian-based RNS markers (N_SNPs_ = 8983; p_SCZ_ threshold < 5 × 10^–8^, Fisher’s exact test *p =* 0.08; OR (CI 95%) = 1.65 (0.88, 2.86); Supplementary Data 3).

Moreover, we described a great overlap between European ancestry based and global RNS markers. Across the 46,872 common LD-independent SNPs in both European and all population RNS data, 1176 out of 2249 RNS markers were among the top 5% with highest probability to be RNS marker (Chi-square test *p <* 1 × 10^–16^).

### Alleles arisen during human evolution and subject to RNS are biased towards protection against schizophrenia

In order to study whether RNS markers confer risk or protection to schizophrenia, we focused on the derived alleles arisen during human evolution (i.e. derived selected alleles). We used LD-independent SNPs with available information of the ancestral allele (the allelic state of the most recent common ancestor of human and the closest primate) in the 1000 Genomes database (N_SNPs_ = 170,609 (98.2% of total LD-independent SNPs)). We calculated GWAS derived-OR_SCZ_ referred to the derived allele and evaluated whether RNS markers associated with schizophrenia were enriched for derived alleles conferring risk or protection to the illness.

**Figure 4:**
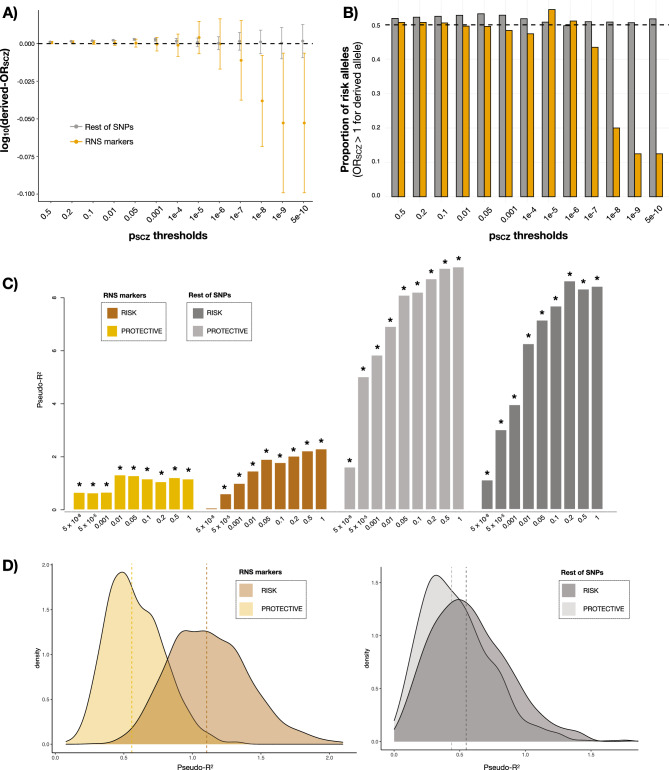
Directionality of RNS enrichment in schizophrenia considering the derived alleles emerged during human evolution. (**A**) Average log_10_(OR_SCZ_) of RNS markers (orange) and the remaining SNPs (grey), referred to the derived alleles that emerged during human evolution, across different SCZ GWAS thresholds. 95% confidence intervals of the means are displayed. (**B**) Proportion of SNPs within RNS markers and the remaining SNPs with a protective or risk effect of the derived allele, across different schizophrenia GWAS thresholds. (**C**) PGS_SCZ_ predictions in the case–control target sample (N_SCZ_ = 1927; N_HC_ = 1561) stratified by RNS. Schizophrenia GWAS summary statistics were subsetted based on the relationship of the SNP to RNS and the risk or protective effect of the derived allele as follows: (i) RNS markers (top 5% of the SNPs with the highest probability of being subject to RNS) whose derived alleles arisen through human evolution confer protection to schizophrenia (derived-OR_SCZ_ < 1; light brown), (ii) RNS markers (top 5% of the most selected SNPs) whose derived alleles arisen through human evolution confer risk to schizophrenia (derived-OR_SCZ_ > 1; dark brown), (iii) SNPs not subject to RNS (from the remaining 95% of SNPs) whose derived alleles arisen through human evolution confer protection to schizophrenia (derived-OR_SCZ_ < 1; light grey), (iv) SNPs not subject to RNS (from the remaining 95% of SNPs) whose derived alleles arisen through human evolution confer risk to schizophrenia (derived-OR_SCZ_ > 1; dark grey). Explained variance attributable to PGS was calculated as the increase in Nagelkerke’s pseudo-R^2^ between a linear model with and without the PGS variable. *P* values were obtained from the binomial logistic regression of SCZ phenotype on PGS, accounting for LD and including sex, age, and 10 MDS ancestry components as covariates. Significant PGS predictions after FDR correction (*p*_*FDR*_ < 0.05) are marked with an asterisk. (**D**) Distribution of variance explained (% pseudo-R^2^) from stratified PGS_SCZ_ predictions based on the abovementioned subsets, using a similar number of variants (N_SNPs_ = 1000 from all available SNPs (P_SCZ_ < 1)) for each analysis. Wilcoxon test was used to compare 1000 pseudo-R^2^ distributions from PGS predictions. See Supplementary Data 7 for detailed results.

We described a significant enrichment of RNS markers for derived alleles with a protective effect on schizophrenia risk at the most stringent GWAS thresholds (average log_10_(derived-OR_SCZ_) (CI95%) = − 0.052 (− 0.099, − 0.006); *p =* 0.031 at P_SCZ_ threshold = 5 × 10^–10^; N_protective-SNPs_ = 7; N_risk-SNPs_ = 1; Fig. 4A,B; Supplementary Data 7A). This enrichment for protective alleles was not observed for derived alleles of SNPs not subject to RNS (average log_10_(derived-OR_SCZ_) (CI95%) = 0.001 (− 0.009, 0.013); *p =* 0.759 at P_SCZ_ threshold = 5 × 10^–10^; N_protective-SNPs_ = 74; N_risk-SNPs_ = 69; Fig. 4A,B; Supplementary Data 7A).

Nine out of 13 RNS markers from schizophrenia GWS *loci* with derived protective alleles have many other pleiotropic effects such as body size, body mass index or systolic blood pressure (Supplementary Data 8). However, this ratio was not significantly greater than for schizophrenia GWS *loci* with derived risk alleles (4 out of 7 RNS markers; Fisher's exact test *P =* 0.47).

Additionally, as pleiotropy might limit effect size^54,55^, we evaluated PGS_SCZ_ performance in the independent CIBERSAM case–control sample (N_SCZ_ = 1927; N_HC_ = 1561) for RNS markers and the rest of non-selected SNPs, by considering variants with protective or derived risk alleles separately (Fig. 4C, Supplementary Data 7B). Since there were different number of risk (N_SNPs_ = 2967) and protective (N_SNPs_ = 3168) SNPs subject to RNS, we then conducted PGS_SCZ_ comparisons after using random permutations of 1000 SNPs for each subset of SNPs (Fig. 4D, Supplementary Data 7B). Across RNS markers, risk variants explained more variance in the SCZ-HC status than protective variants do (Wilcoxon test; *p <* 10^–16^; median R^2^_RISK_ = 1.10; median R^2^_PROTECTIVE_ = 0.54). The difference in explained variance between risk and protective variants was smaller across non-RNS SNPs (Wilcoxon test; *p =* 4.5 × 10^–13^; median R^2^_RISK_ = 0.54; median R^2^_PROTECTIVE_ = 0.46). We observed similar MAF across protective (MAF(CI95%) = 0.366 (0.358, 0.374)) and risk (MAF(CI95%) = 0.336 (0.329, 0.344)) RNS markers, thus ruling out that the results could be inflated by higher MAF of risk SNPs.

### Functional enrichment of schizophrenia GWS *loci* subject to RNS

**Figure 5:**
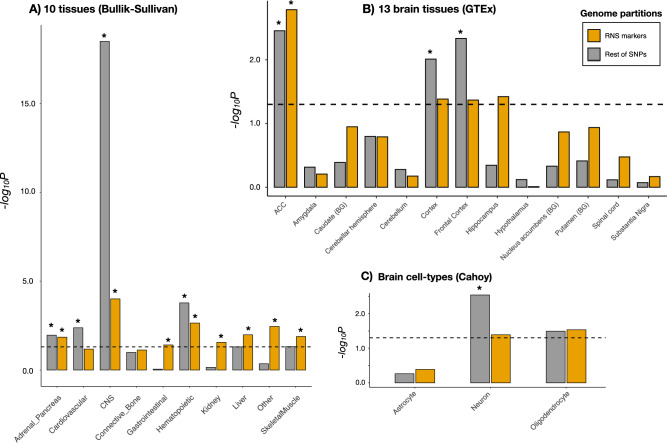
Comparative enrichments of tissue and cell-type signatures across RNS markers and the remaining non-selected SNPs. Tissue and cell-type sub-annotations were created from the intersection between the described annotations (RNS markers and the rest of SNPs) and gene expression data from 10 whole tissues^56^, 13 brain-related tissues (Brain GTEx^57^), and 3 brain cell-type annotation files (neurons, astrocytes, and oligodendrocytes^57,58^). See Methods for further details. (**A**) Heritability enrichment of schizophrenia across RNS markers and the rest of non-selected SNPs within genes expressed in 10 whole tissues^56^. *P* values are derived from one-sided t-tests evaluating whether the cell-type enrichment within a particular tissue sub-annotation is higher than the control background annotations. We considered all RNS markers and the rest of the SNPs from schizophrenia GWAS control annotations since they represent the genomic background in which tissue sub-annotations are embedded. (**B**) Heritability enrichment of schizophrenia across RNS markers and the rest of the non-selected SNPs within genes expressed in 13 brain-related tissues (Brain GTEx^57^). To control for brain expression bias, enrichments across brain-tissue sub-annotations were compared to “anti-target tissue” sub-annotations. The latter were created as the intersection between the RNS or the rest of SNPs’ annotations and the remaining 12 brain-related tissue annotations. *P* values are from one-sided t-tests evaluating whether the enrichment within a particular brain tissue sub-annotation is higher than the anti-target brain tissue sub-annotation. (**C**) Heritability enrichment of schizophrenia across RNS markers and the rest of the non-selected SNPs within genes expressed in 3 brain cell-type annotation files (neurons, astrocytes, and oligodendrocytes^57,58^). To control for brain expression bias, enrichments across cell-type sub-annotations were compared to “anti-target cell-type” sub-annotations. The latter were created as the intersection between the RNS or the rest of SNPs’ annotations and the remaining 2 cell-type annotations. One-sided t-tests were performed to evaluate whether the enrichment within a particular brain cell-type sub-annotation was higher than the anti-target brain cell-type sub-annotation. Dashed lines represent the nominal significance threshold (*p <* 0.05). * Significant results after Benjamini–Hochberg FDR correction. ACC: Anterior cingulate cortex; BG: Basal ganglia; CNS: central nervous system.

We finally aimed to describe the functional characteristics of the *loci* that were both subject to RNS and associated with schizophrenia. We used LDSC applied to specifically expressed genes (LDSC-SEG) to evaluate the schizophrenia heritability enrichments across RNS markers within 10 different tissues, 13 brain related tissues, and 3 brain cell-types and compared them to the LDSC-SEG enrichments across the rest of SNPs (Supplementary Data 4). While schizophrenia predisposing variation non-subject to RNS was found to be enriched at cortical brain tissue (FDR-*p =* 0.029) and neuronal cell-type (FDR-*p =* 0.017), schizophrenia predisposing variation within RNS markers was found to be enriched beyond brain-related tissues and not enriched in any brain-related cell types (Fig. 5, Supplementary Data 4).

We also characterized RNS signals by comparing genes mapped from schizophrenia GWS *loci* subject and not subject to RNS. Using fine mapping results from the latest SCZ GWAS^49^, we considered the broad set of 628 mapped from GWS loci with low numbers of expected causal SNPs (K < 3.5; see Methods, Supplementary Data 9A). Genes mapped from GWS *loci* harboring (22 genes) and not harboring (468 genes) RNS markers were tested for functional over-representation analysis (ORA) using the whole gene-set as background. Although no brain-relevant overrepresentation was found in any case, we observed an enrichment of *loci* subject to RNS in brain unrelated functional signatures (miRNA metabolic process p_FDR_ = 0.006; gland development p_FDR_ = 0.026; epidermis development p_FDR_ = 0.026; Supplementary Data 9B).

## Discussion

Here in this work, we characterized the RNS signatures that occurred after the human diaspora out of Africa across the whole genome and demonstrated a significant enrichment of RNS markers across schizophrenia predisposing *loci* as well as a significant enrichment in their contribution to schizophrenia heritability. Then, by analyzing the derived and ancestral alleles we observed that RNS markers were enriched for derived alleles conferring protection to schizophrenia. This tendency towards protective versus derived risk alleles was not found across variants not subject to RNS, thus suggesting a positive selection of protective schizophrenia alleles during recent evolution of human populations. The biological characterization of schizophrenia predisposing *loci* subject to RNS and their mapped genes showed an underrepresentation of brain and neuronal related functions as well as more pleiotropic associations relative to schizophrenia predisposing *loci* not subject to RNS. These results suggest that recent selection of schizophrenia alleles could be promoted by conferring selective advantage through other non-psychiatric phenotypes. Our results suggest pleiotropy as a likely mechanism behind RNS that could explain, at least in part, the recent evolutionary dynamics of schizophrenia predisposing genetic variation.

Previous studies have tried to explain why genetic predisposing variation to schizophrenia persists in the population, despite the reproductive fitness reduction in affected individuals^20,21,24^. In this sense, many authors have described the persistence of schizophrenia predisposing alleles as a result of balanced selection or because they could provide fitness advantages in certain environments^22,41,59,60^. However, in this study, we observed that schizophrenia GWS *loci* are enriched in variants that have rapidly expanded across modern human populations (and are therefore subject to RNS) and that the derived alleles confer protection rather than risk to schizophrenia. Thus, our results are in line with recent studies reporting negative selection of schizophrenia predisposing alleles^26,61^. Pardiñas et al.^26^ have suggested a plausible explanation of the purported schizophrenia evolutionary paradox of persistent common variation and progressive removal of risk alleles through background selection^62^. By this mechanism, removal of rare haplotypes harboring deleterious mutations could reduce genetic diversity and allow common alleles with small deleterious effects to maintain a high frequency by drift^26,62^.

Apparent contradictory results regarding the positive or negative selection of alleles that predispose to schizophrenia need to be integrated using a dynamic perspective. Since our study has focused only on genetic selection initiated after African populations started their dispersion into Eurasia around 60,000–100,000 years ago^31–33^, a reliable explanation for the persistence of common alleles conferring risk to schizophrenia may reside on fitness advantages provided during earlier stages of the human evolution. In this sense, a previous study focusing on the Sapiens-Neanderthal divergence described a pattern of positive selection of schizophrenia predisposing variation that could have contributed to the *Homo sapiens* success against other human species^41^. Moreover, schizophrenia *loci* were also described to be enriched in genomic regions that had experienced specific evolutionary acceleration during early human evolution (HARs) in comparison with other non-human primates^63^. Although these evidences reinforce the idea of schizophrenia predisposing variation acquisition as a price to pay for human abilities such as language development^24^ or even more adaptive immunological profiles^64^, the evolutionary pressures to retain schizophrenia risk alleles as an evolutionary advantage might have stopped acting before the dispersion of the *Homo sapiens* from Africa. In line with our results, studies exploring RNS patterns have not found a pattern of positive selection of schizophrenia predisposing alleles^26,27,29^. A recent study has proposed an evolutionary framework by which schizophrenia risk alleles increased their frequency with the development of the social brain and high-order cognitive functions, but after a turning point the trend was reversed and a negative pressure against schizophrenia alleles became the standard^61^. Interestingly, this turning point from positive to negative selection of predisposing alleles in schizophrenia could be shared by other psychiatric traits. In fact, a recent work observed a similar scenario for attention-deficit/hyperactivity disorder (ADHD): while introgressed Neanderthal alleles were enriched in ADHD risk variants, modern environments triggered the progressive elimination of ADHD-predisposing alleles^65^. This temporal perspective should also prevent readers from reaching conclusions from current evolutionary dynamics. While our approach explored RNS from around 100,000 years ago, recent methods have been used to assess modern selection from up to 100 generations (around 2000 years ago)^66,67^. In these recent studies, schizophrenia was also affected by even more recent negative selection^67^.

Given the evidence here described about the relationship between predisposing variation to schizophrenia and RNS throughout human populations, we assessed whether these evolutionary dynamics were also present at the Asian or European intrapopulation level. While the association between RNS and the genetic predisposition to schizophrenia is replicated in European populations, this association appears to be absent among East Asian populations. This finding suggests that the observed relationship between schizophrenia and recent natural selection may be driven by selective pressures resulting from other traits, acting through specific environments that are unique to certain populations. However, the considerably smaller sample size of the Asian schizophrenia cohorts compared to the European samples could be the main cause of the lack of association between recent natural selection and schizophrenia predisposing *loci*.

Upon assessing the whole SNP-based heritability enrichment across tissue and cell-type genome annotations, stratified by RNS markers, our results demonstrate a depletion of brain and neuronal-related functions in *loci* subject to RNS, as compared to those not subject to RNS. While common schizophrenia predisposing variation not subject to RNS exhibit clear patterns of heritability enrichment in brain-related tissues and neuronal cell types, aligning with expectations from prior studies, schizophrenia predisposing variation under RNS display lower enrichments in neuronal and brain-related functions, along with significant heritability enrichments in other tissues not directly linked to the central nervous system, such as the kidney or liver (Fig. 5). Similarly, functional enrichment analyses of fine-mapped genes derived from RNS-affected SNPs reveal enrichment in biological functions unrelated to the brain.

In fact, our results pointing to pleiotropic effects of schizophrenia *loci* under selection are in line with previous findings on specific schizophrenia related genes with marked evolutionary patterns. For instance, the GWS schizophrenia risk variant rs13107325 (C/T) within the *SLC39A8* gene has been described to be under positive selection of its derived risk T allele in Europeans^36,39^. This evolutionary event, however, has been reported to be driven by the migration of modern humans out of Africa and the consequent adaptation to colder climates by reducing blood pressure and the risk for hypertension^22,40^. Similarly, survival of the derived risk T allele of variant rs1150711 (C/T) of the *ZNF323* gene could be the result from the ameliorated lung function provided by this allele to European populations^59^. These and similar examples of antagonistic pleiotropy, in which the allele that predisposes to schizophrenia confers adaptive advantage or protects against another condition, have been used to explain the evolutionary paradox of schizophrenia. However, our study suggests that, on a genome-wide scale, non-antagonistic pleiotropy could be contributing to the RNS enrichment across schizophrenia common variation. In this sense, recent positive selection of schizophrenia derived protective alleles could also be a likely by-product of the pleiotropic effect of some genetic variants favoring adaptation to the environment during modern evolution of human populations^68,69^.

Differing genetic risks for schizophrenia described across distinct populations^29^ could therefore be the result of distinct evolutionary pressures related to other phenotypes, with predisposing variants having also an impact on schizophrenia risk. Although most of schizophrenia alleles have been described to exert similar effects across populations^70^, allele frequencies could vary across populations, thus leading to differences in individual genetic risk profiles. In this sense, greater polygenic scores for schizophrenia have been described in African populations^29,71^. However, reportedly greater polygenic scores and our finding of positive selection of schizophrenia derived protective alleles do not imply that African populations are at higher genetic risk for schizophrenia. Since GWAS have been performed mainly in cohorts of European ancestry, there is a limited portability for the estimation of polygenic risk scores across African cohorts using the available GWAS data^65,71^, and these inferences should be avoided.

By evaluating the PGS predictions on schizophrenia within RNS with derived protective and risk alleles separately, we observed greater variance explained across similar numbers of risk than protective RNS markers (Fig. 4D). These findings suggest that risk variants under RNS are more likely to increase schizophrenia risk by acting in a cumulative way. It has been suggested that strong negative selection acting against highly pleiotropic deleterious variants of large effect may give rise to common pleiotropic variants of lower effect sizes^54,55^. Consequently, the predictive performance of a set of variants will decrease as the level of pleiotropy increases. If there is an excess of pleiotropic variants among the RNS derived protective alleles compared with derived risk alleles, this might explain the higher predictive capacity of a PGS based on derived risk alleles.

This study was subject to several limitations. First, we described RNS markers using whole 1000 Genomes data, while we used association data from mainly European-ancestry schizophrenia cohorts (around 80%^49^). In this sense, however, our sensitivity analyses considering population specific data lead to similar results in the case of European population, and therefore validating our findings, although this scenario was not replicated in East Asian populations. Therefore, the implications of our results suggesting protective selection by non-antagonistic pleiotropy may be limited, since environments shaping this evolutionary pattern could be absent in other populations. Nevertheless, this inconsistency between European and East Asian population specific analyses may be caused by a great difference in statistical power, and larger Asian cohorts should be used to rule out this possibility. Second, to have sufficient statistical power, our analysis is restricted to variants having a MAF higher than 5%. This filter could have removed many GWAS signals with remarkable RNS effects. For instance, a variant within *SLC39A8,* a schizophrenia-associated gene with well-described evolutionary properties, is absent from this study due to its low allele frequency. Third, along the lines of a previous related study^72^, we have used the top 5% markers with the highest probability of being subject to RNS for our main analyses in order to have enough statistical power. Finally, it is worth noting that while pcadapt has been employed in previous studies involving similar 1000 Genomes data^47^, PCA-based methods have been reported to have a susceptibility to false positives when applied to populations with pronounced stratification and a non-continuous principal component space. In this regard, the concurrence of selection outliers detected by alternative *Fst* methods served as validation for our identification of RNS markers using pcadapt.

These limitations notwithstanding, our results shed additional light on the relationship between RNS and schizophrenia. By taking advantage of the genome wide scans and statistical outputs provided by novel methods such as *Pcadapt*, we have described a clear enrichment of protective RNS markers across schizophrenia GWS loci and suggest non-antagonistic pleiotropy as a likely explanation. This novel perspective could help to integrate previous contradictory findings related to the evolutionary paradigm of schizophrenia and pave the way for further studies of the evolutionary patterns of other neuropsychiatric disorders and human behavioral traits.

## Methods

### RNS scans in human populations

To evaluate the selective pressures that took place after the human diaspora out of Africa (from ca. 100,000 years ago to present), we performed scans detection of RNS signatures using a principle components-based approach implemented in *Pcadapt* v 4.3.2^47,48^. *Pcadapt* performs PCA and computes *p*-values to test for the presence of selection outliers, based on the correlations between genetic variation and the selected K principal components. Briefly, for a given SNP, z-scores are obtained by regressing the SNP position on the K principal components. The test implemented is based on the multi-dimensional Mahalanobis distance from the SNP to the K components, thus describing how distant the SNP is from the mean.

To study RNS signatures we used genetic data from the 1000 Genomes phase 3 sequencing database^73^, across African, European, and Asiatic populations. American populations, who show greater genetic heterogeneity due to recent admixture events, were eliminated from the analysis, as suggested by the *Pcadapt* developers^47^. Moreover, given the elevated inbreeding coefficients for some of the 10,000 Genomes subpopulations^74^, only 1699 subjects that have been previously described as outbred and unrelated^74^ were finally considered. We retained common genetic variation (MAF > 0.05), biallelic, and overlapping with summary data from the schizophrenia GWAS used in this study, thus yielding a total of 5,554,437 SNPs.

Since LD can affect ascertainment of population structure^75^, *Pcadapt* accounts for LD-genome structure (window size = 200 SNP, r^2^ = 0.1) for the estimation of RNS probabilities for each of the 5,554,437 SNPs considered. The distribution of PC loadings was evaluated to ensure that selection signals correspond to regions subject to adaptation rather than to regions of lower recombination (high LD). We used Cattell’s rule to select the appropriate number of PCs (K = 3). *Pcadapt* test was then performed and derived *p*-values (*p*_*RNS*_) for every SNP were calculated to inform about their likelihood of being subject to RNS.

Throughout the subsequent analyses of the study, we consider the top 5% selected markers (5% of SNPs with the lower *p*_*RNS*_*;* RNS markers) after LD-clumping based on schizophrenia GWAS *p*-values (PLINK v1.9 *parameters-clump-r2 0.1-clump-kb 500*) and refer to them as RNS markers. We prioritized the association with schizophrenia and the top 5% RNS markers were selected to have enough SNPs with which to perform the subsequent analyses. However, to ensure that the reported results were not biased by LD-clumping based on SCZ *p*-values, we also considered the top 5% RNS markers after LD-clumping based on *p*_*RNS*_ and refer to them as LD-RNS markers.

We also used OutFLANK, that provides a robust estimation of the null distribution of a *Fst* test statistic^76^, between African and European 1000 thousand genomes populations, as an additional selection parameter to compare with *Pcadapt* estimates.

### Samples and GWAS summary data

We used the latest summary statistics of the schizophrenia GWAS (PGC-SCZ3) conducted by the Psychiatric Genomics Consortium (PGC)^49^ (data available at https://www.med.unc.edu/pgc/results-and-downloads). Main analyses were performed using the whole summary statistics (67,390 schizophrenia/schizoaffective disorder cases and 94,015 controls). Moreover, both ancestry-specific summary statistics for European (53,386 cases and 77,258 controls) and East Asian subjects (14,004 cases and 16,757 controls) were used for additional sensitivity analyses to explore association between RNS signals within European or East Asian populations and ancestry-specific predisposing variation to schizophrenia. As a specificity analysis for the detection of selection signals, we also used Alzheimer’s disease^51^ GWAS summary statistics in independent analyses. This disorder was selected based on its adequate statistical power comparable to that of schizophrenia, its described clinical similarities, and its later age at onset, after natural selection pressure has exerted its effect. Details regarding the summary statistics used are described in Supplementary data 10.

The Major Histocompatibility Complex (MHC) region was excluded in all analyses and only biallelic SNP and those with an imputation quality score > 0.9 were considered. Overall, 5,554,437 variants from 1000 Genomes overlapping with schizophrenia summary data were converted to PLINK format and used as the input for *Pcadapt* in the analysis. 6,693,073 variants were used in the case of Alzheimer’s disease.

We used a case–control sample including 1927 schizophrenia cases (65% males) and 1561 healthy controls (HC) (55% males) from CIBERSAM (Centro de Investigación Biomédica en Red en Salud Mental, Spain) as an independent target sample for PGS predictions (SCZ_CIBERSAM). All research was performed in accordance with relevant guidelines/regulations. Informed consent was obtained from all individual participants included in the study, and ethic boards/committees from the following involved Spanish hospitals approved the protocol: Clinical Research Ethics Committee of the Hospital Sant Joan de Reus, Research Ethics Committee of Asturias, Research Ethics Committee of Cantabria, Bioethics Commission of the University of Barcelona (CBUB), Galician Regional Research Ethics Committee, Scientific Research Ethics Committee of Hospital Gregorio Marañón and Research Ethics Committee of the Valencia’s clinical Hospital. All participants were genotyped as part of the Psychiatric Genomics Consortium (PGC), and passed quality control (QC filters) per PGC-SCZ2 criteria^39^. There is no overlap between PGC-SCZ2 and SCZ_CIBERSAM samples.

### Polygenic score (PGS) analyses

PGS for schizophrenia (PGS_SCZ_) were calculated from PGC-SZ2^39^ GWAS summary statistics as discovery sample and SCZ_CIBERSAM case–control cohort as target sample. Summary data from the latest schizophrenia GWAS^49^ was not used to estimate PGS because target sample was included as part the cohorts in the study. PGS_SCZ_ were calculated using SNPs present in the 1000 genomes database for the studied populations. We included only biallelic variation, with imputation quality scores > 0.9, and excluded indels. 65PLINK 1.9 was used to calculate PGS across schizophrenia patients and healthy controls weighted by the logOR in the discovery sample. Standardized PGS were calculated, and significance was evaluated by logistic regression, using case–control status as dependent variable and sex, age, and the first 10 MDS ancestry components as covariates. We calculated explained variance attributable to PGS as the increase in Nagelkerke’s pseudo-R^2^ between a model with and without PGS variable.

Initially, we used several P thresholds (*P <* 5 × 10^–8^, 5 × 10^–5^, 1 × 10^–3^, 0.01, 0.05, 0.1, 0.2, 0.5 and 1) and the whole genome variation to estimate predictions in the SCZ_CIBERSAM case–control cohort. The most significant P threshold (*P <* 0.2) was selected for the subsequent PGS analyses stratified by RNS. We then performed stratified PGS predictions to compare SNPs that were subject to RNS to those that were not. Summary SNP data with P_SCZ_ < 0.2 was divided into 20 quantiles of increasing *p*_*RNS*_. PGS predictions were performed across the 20 SNP quantiles in the CIBERSAM case–control sample. Linear regression was performed to evaluate the change in the variance explained by PGS on case–control status in the CIBERSAM sample across the 20 quantiles. Nagelkerke’s pseudo-R^2^ from PGS predictions using the first quantile, corresponding to the 5% of SNPs with the highest probability of being subject to RNS, was compared against the distribution of pseudo-R^2^ obtained by selecting the same number of SNPs from the remaining 95% of SNPs. On account of the likely bias in prediction due to the non-random genome features of SNPs subject to RNS, selection of SNPs from the remaining 95% was carried out by identification of sets of randomly drawn SNPs that were matched to the top 5% selected SNP based on allele frequency, number of SNPs in LD, distance to nearest gene, and gene density using default parameters from SNPsnap^77^ (https://data.broadinstitute.org/mpg/snpsnap/). Since an earlier study by Price et al. suggested that recent selection signals could be partly explained as artifacts caused by long-range LD regions^53^ that can lead to inflated variance explanations from variants subject to RNS, we repeat the previously explained stratified PGS predictions after removing 24 described Long-Range LD Regions as a sensitivity analysis to assess whether the results hold.

Additionally, we also calculated stratified PGS after subsetting SNP summary data into those for which the derived allele that emerged during human evolution conferred protection (derived-OR_SCZ_ < 1) or risk (derived-OR_SCZ_ > 1) to schizophrenia. Across variants subject (RNS markers) and non-subject to RNS, independently, we compared 1000 predictions from protective and risk variants, using a similar number of SNPs (N_SNPs_ = 1000), on the SCZ_CIBERSAM case–control sample. Wilcoxon test was used to compare the pseudo-R^2^ distributions of predictions for SNPs with a protective (derived-OR_SCZ_ < 1) or risk (derived-OR_SCZ_ > 1) derived allele across SNPs subject (RNS markers) and non-subject to RNS.

### Partitioning heritability (LDSC)

We calculated SNP-based heritability (*h*^*2*^_*SNP*_) estimates for the resulting genome partitions after dissections of schizophrenia summary genetic data based on RNS markers following the recommended procedure^15,57^ (https://github.com/bulik/ldsc/wiki/Partitioned-Heritability).

First, we created per SNP annotation files (one per chromosome and desired annotation). Each file consisted of a row per SNP and a column for each sub-annotation (1 = a particular SNP is part of that sub-annotation, 0 = The SNP does not belong to the annotation). Annotation files were created for:

i) RNS markers (SNPs from schizophrenia GWAS (PGC-SCZ3) summary statistics^49^ that belong to the top 5% with the highest likelihood (lowest *p*_*RNS*_) of being a RNS marker) and the rest of the SNPs (the remaining 95% of the SNPs from schizophrenia GWAS summary data).
ii) Sub-annotations from the intersection between the described annotations (RNS markers and the rest of SNPs) and gene expression data from 10 whole tissues^56^, 13 brain-related tissues (Brain GTEx^57^), and 3 brain cell-type annotation files (neurons, astrocytes, and oligodendrocytes^57,58^).

We used *ldsc v1.0.1*^15,57^, a command line tool for estimating heritability. We performed both heritability enrichment analyses across the described annotations (*–h2*) and one-sided t-tests to evaluate whether the cell-type enrichment in schizophrenia within a particular annotation was higher than the same cell-type enrichment in schizophrenia outside the target annotation (anti-target) but within a background annotation (using *–h2-cts*). Control background annotations from the original study were used^56,57^.

We ran LDSC using associated data files from phase 3 of the 1000 Genomes Project^73^. LD scores were computed for each annotation file using the recommended parameters: 1-cM window (–*ld-wind-cm* 1), restriction to Hapmap3 SNPs, and exclusion of the MHC region due to its high gene density and exceptional LD, as recommended by the developers^15^. The ‘–*overlap-annot’* argument and 1000 Genomes phase 3—based frequency files (‘1000G_Phase3_frq’ files via *–frqfile-chr* argument) and LD weights (‘*weights_hm3_no_hla’* files via*-w-ld-chr* argument) were used for LD score calculations.

Partitioned LDSC computes the proportion of SNP heritability associated with each annotation column while considering all the remaining annotations. This is performed by regression models using the estimated LD-scores jointly with other independent LD scores for baseline annotations to improve the model performance. We used the full baseline model v2.2, consistent of a full annotation column (1 per all SNPs) and 158 independent functional annotations, available at LDSC repository (https://data.broadinstitute.org/alkesgroup/LDSCORE/), as independent LD scores.

### Characterization of schizophrenia genome-wide significant (GWS) *loci* with and without RNS markers

We used FINEMAP results of the schizophrenia GWS *loci* from the latest schizophrenia GWAS^49^, and compared the functional overrepresentation of mapped genes from GWS loci harboring RNS markers against those genes not harboring RNS markers. Again, the MHC region was not considered due to its complex LD structure. In order to have sufficient statistical power, the broad fine-map set 628 genes (435 protein-coding) that contained at least one credible SNP from 249 regions with low numbers of expected causal SNPs (K < 3.5) were used^49^. Genes mapped only from RNS markers (22 genes) were compared against genes mapped by SNPs not subject to RNS (468 genes).

Functional overrepresentation of genes from each category was assessed by over-representation analysis (ORA) with WEB-based GEne SeT AnaLysis Toolkit (WebGestalt; http://www.webgestalt.org/)^78^. All mapped genes from schizophrenia GWS *loci* studied here in this study were used as a background list. Enrichment across gene sets from Gene Ontology (GO) cellular components (CC) and biological functions (BF) categories was evaluated. FDR by Benjamini–Hocheberg adjustment was used to evaluate enrichment significance across GO gene sets, and a minimum threshold of 5 genes overlapping with each gene set was considered.

### Statistical analyses

Statistical analyses were performed using R (https://www.r-project.org/). One-sample Kolmogorov–Smirnov test was performed to evaluate the overrepresentation of SNPs subject to RNS after principal components-based approach implemented in *Pcadapt.* Fisher’s exact tests were performed to study the enrichment for the RNS markers ( top 5% RNS *loci*; LD-based on *p*_*SCZ*_; N_SNPs_ = 8679) or the LD-RNS markers (top 5% RNS *loci*; LD-based on *p*_*RNS*_; N_SNPs_ = 6262) across the different schizophrenia GWAS thresholds (*p*_*SCZ*_ < 5 × 10^−8^, 1 × 10^−7^, 1 × 10^−6^, 1 × 10^−5^, 1 × 10^−4^, 0.001, 0.01, 0.05, 0.1, 0.2, and 0.5). We also analyzed enrichment for the RNS markers for Alzheimer’s disease (N_SNPs_ = 6263). Spearman correlation between *P* values from GWAS and RNS was performed (− log_10_ *p*_*GWAS*_, vs. − log_10_ *p*_*RNS*_). Correlations were confirmed by conducting 10,000 random permutations of the GWAS results with respect to the *p*_*RNS*_ values and testing whether the observed correlation coefficients were significantly higher than the ones in the null distribution of the permuted datasets.

We calculated GWAS derived-OR_SCZ_ (referred to the derived allele) for each SNP and evaluated the overrepresentation of protective or derived risk alleles across schizophrenia GWAS thresholds: chi-square tests were performed to compare the proportion of derived risk alleles (derived-OR_SCZ_ > 1) within and outside RNS markers (top 5% RNS *loci*) across schizophrenia GWAS thresholds (*p*_*SCZ*_ < 5 × 10^–10^, 1 × 10^–9^, 1 × 10^–8^, 1 × 10^–7^, 1 × 10^–6^, 1 × 10^–5^, 1 × 10^–4^, 0.001, 0.01, 0.05, 0.1, 0.2, and 0.5). Additionally, one sample t-tests were performed to compare the average derived-OR_SCZ_ within and outside RNS markers (top 5% RNS *loci*) to the neutrality across schizophrenia GWAS thresholds (we consider neutrality, i.e. no risk or protection bias is found when the confidence interval includes the value derived-OR_SCZ_ = 1). Two sample t-tests were also performed to compare derived-OR_SCZ_ within vs outside RNS markers.

We used Fisher's exact tests to evaluate the enrichment of schizophrenia GWS *loci* subject to RNS with the derived protective or risk allele having the lead association with schizophrenia among all phenotypes included in GWAS Atlas (https://atlas.ctglab.nl/)^79^.

### Supplementary Information

Supplementary Figures.

Supplementary Information 1.

## Data Availability

The results generated during and/or analysed during the current study are available from the corresponding author on reasonable request.
