# Supplementary material for: Recent natural selection conferred protection against schizophrenia by non-antagonistic pleiotropy
Source: Sci Rep. 2023 Sep 19;13:15500. doi: 10.1038/s41598-023-42578-0 (PMC10509162; doi:10.1038/s41598-023-42578-0)

Recent natural selection conferred protection against schizophrenia by non-antagonistic pleiotropy

SUPPLEMENTARY FIGURES

Supplementary Figure 1. Scree plot displaying the percentage of variance explained by each PC **2**

Supplementary Figure 2. Principal components analysis (PCA) on 1000 genomes populations **3**

Supplementary Figure 3. Genome-wide display of RNS loci across the genome **4**

Supplementary Figure 4. Distribution of SNP studied accumulation p_{RNS} quantiles.......**5**

Supplementary Figure 5. Visual evaluation for the presence of recent natural selection outliers (Q-Q plot) **6**

Supplementary Figure 1. Scree plot displaying the percentage of variance explained by each PC. The number of PCs selected corresponds to the point where the scree plot is a steep curve followed by a bend and a straight line (Cattell's rule). Here, K = 3 is the optimal choice for K.

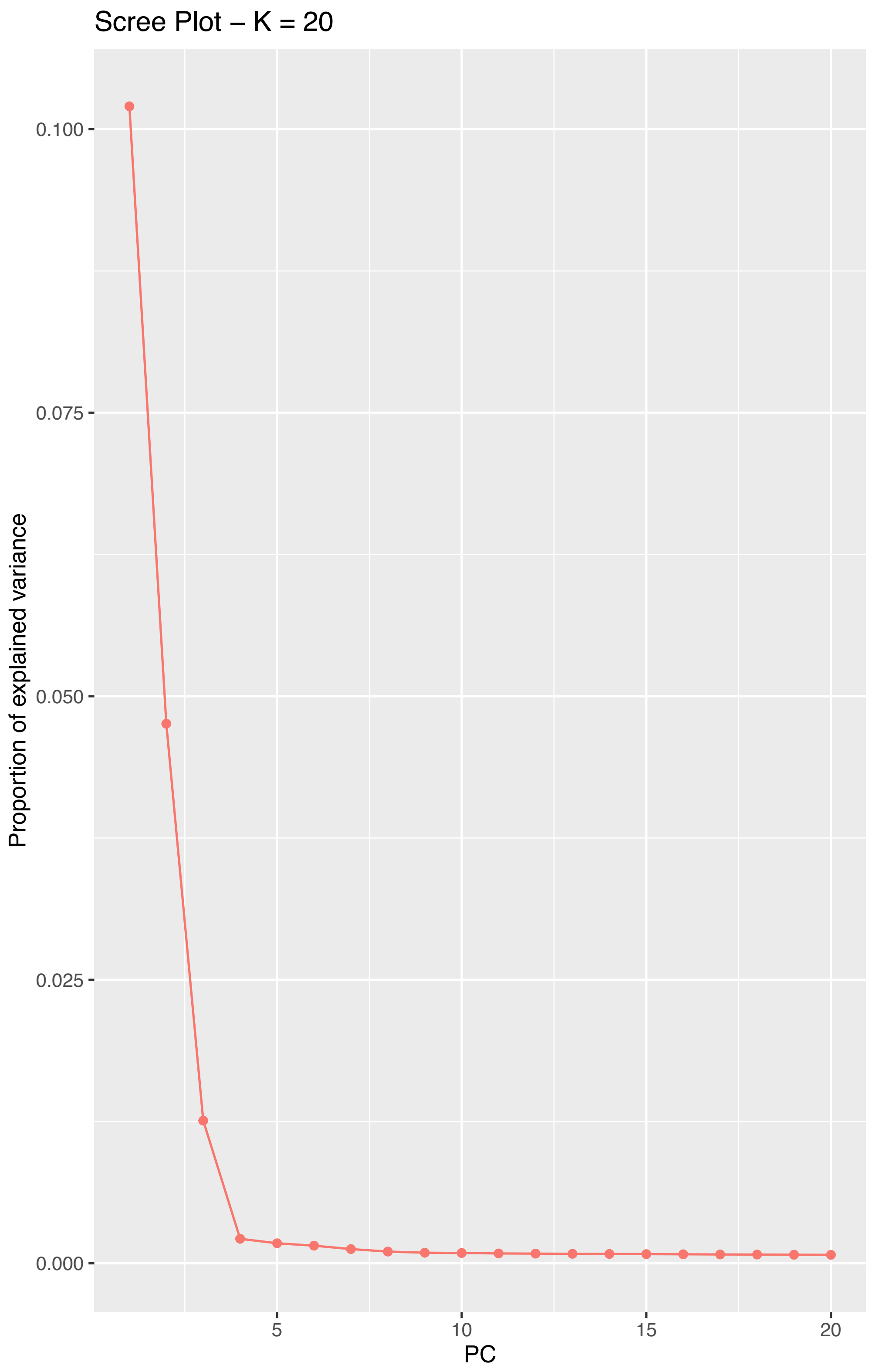

Supplementary Figure 2. Principal components analysis (PCA) on 1000 genomes populations. Score plot from PCA describing population structure of the subjects included in the study. 3 principal components were chosen based on Cattell's rule (**Supplementary Figure 1**). 1000 genomes phase 3 sequencing data 3 across African, European and Asiatic populations were used. We retained common biallelic genetic variation (MAF > 0.05), overlapped with summary data from the SCZ GWAS used in this study and with high imputation quality (score > 0.9). A total of 5,220,878 SNPs were used.

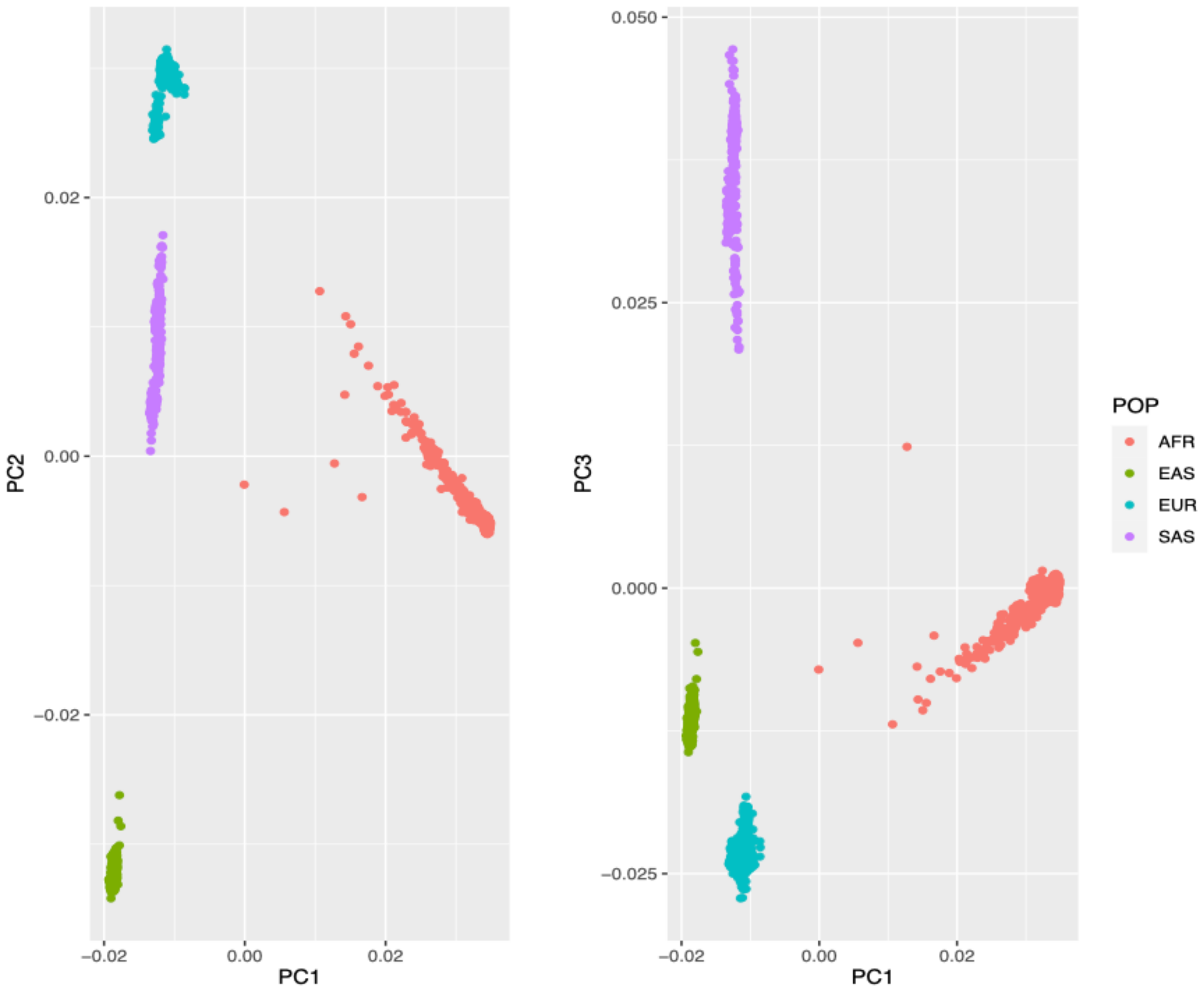

Supplementary Figure 3. Genome-wide display of RNS loci across the genome. Manhattan plot displaying probability to be a RNS signal ($-\log_{10}(p_{RNS})$) across autosomes is shown. 1000 genomes phase 3 sequencing data 3 across African, European and Asiatic populations were used. We retained common biallelic genetic variation (MAF > 0.05), overlapped with summary data from the SCZ GWAS used in this study and with high imputation quality (score > 0.9). A total of 5,554,437 SNPs were used. In the estimation of p_{RNS} for all SNP data, LD-genome structure (window size =200 SNP, r^2 = 0.1) was considered by pcadapt to avoid bias in PCA.

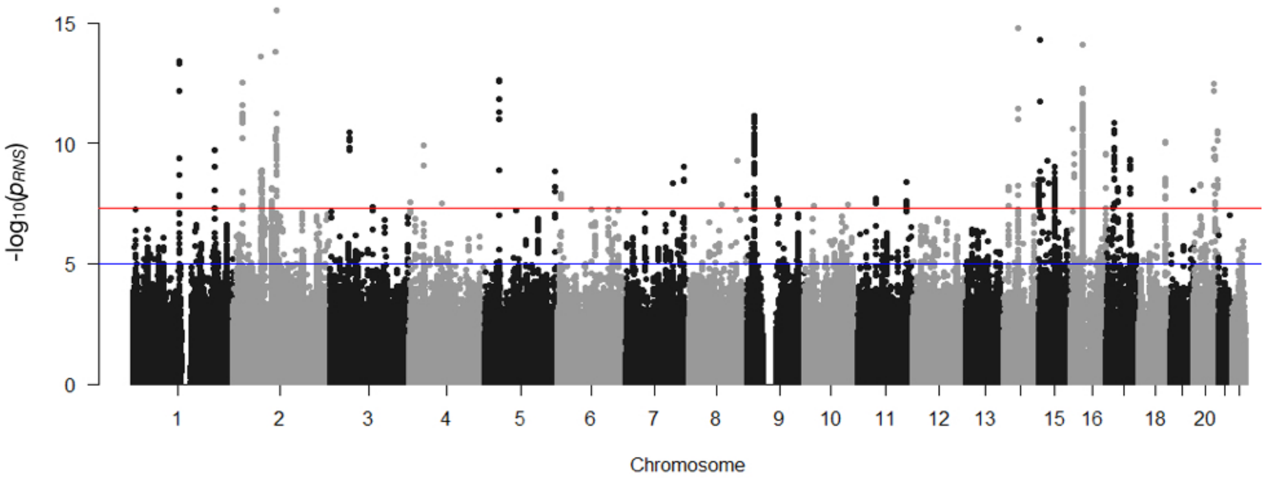

Supplementary Figure 4. Distribution of SNP studied accumulation p_{RNS} quantiles. Histogram of p_{RNS} indicating the higher accumulation of SNP with low p_{RNS} values than expected by chance.

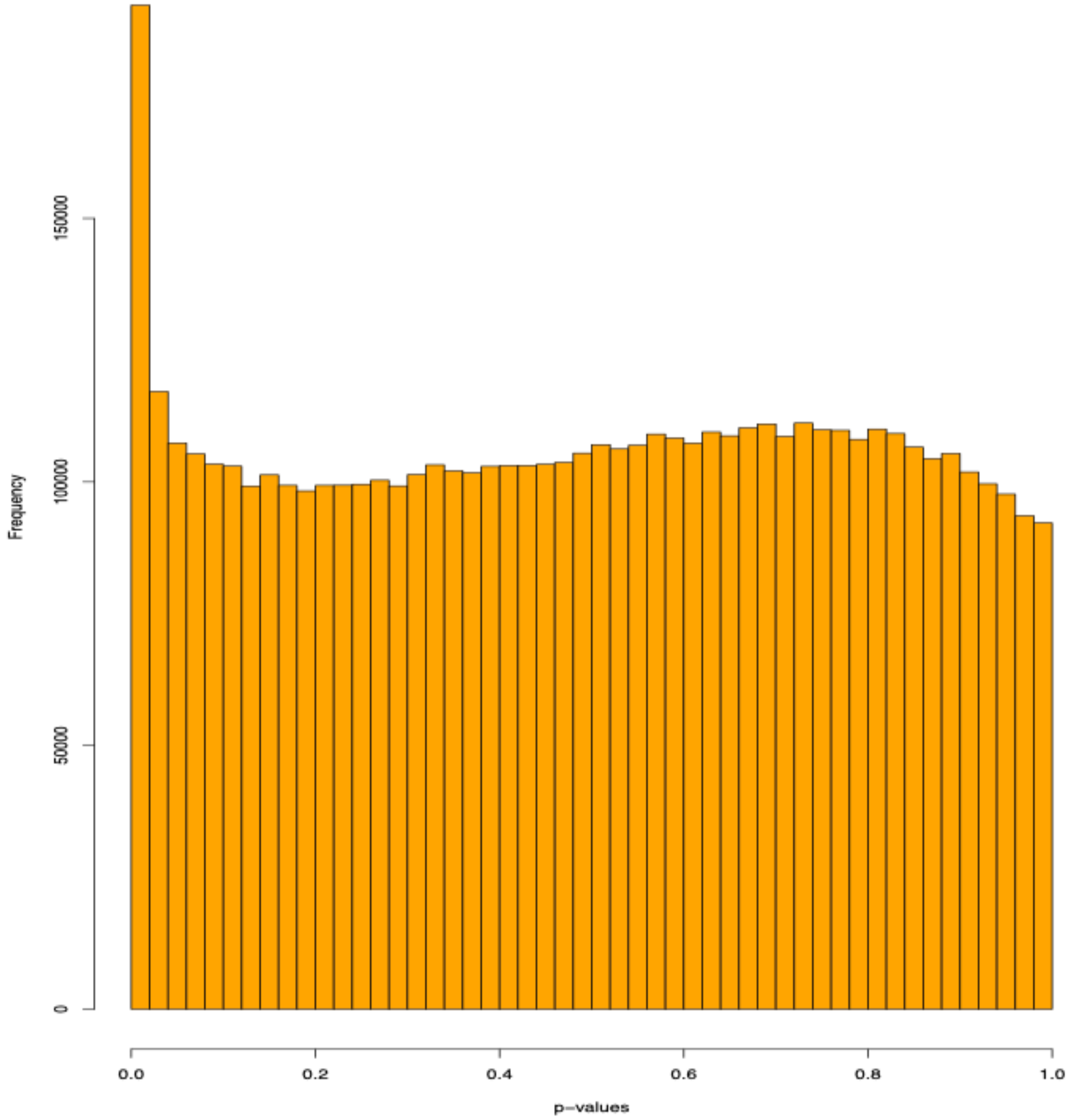

Supplementary Figure 5. Visual evaluation for the presence of recent natural selection outliers (Q-Q plot). A) Q-Q plot comparing the expected vs observed p-values for the SNP here studied. B) Statistical distribution of the Mahalanobis distance between each SNP and the PCs (K = 3).

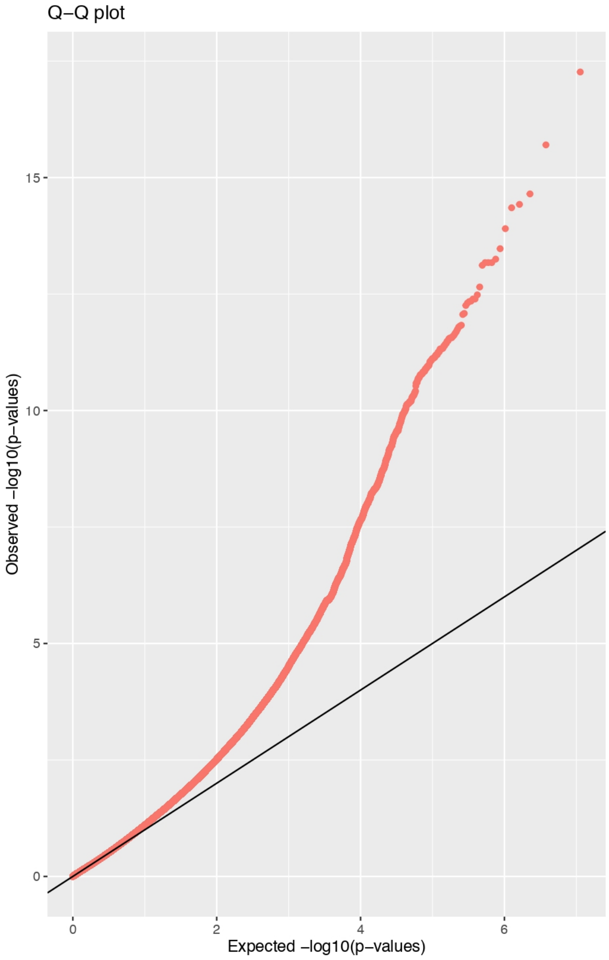

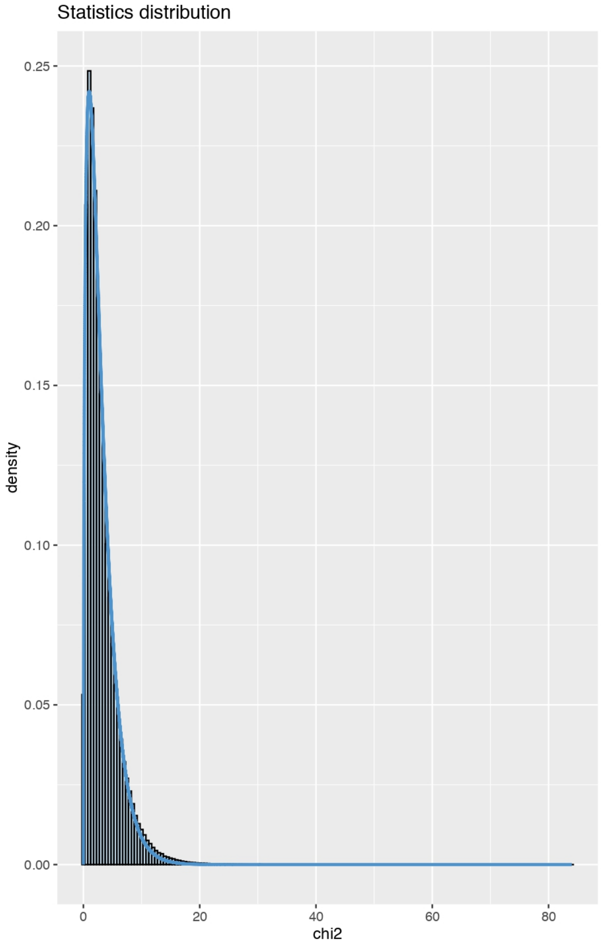

Supplement: Supplementary file 1 — Supplementary Figures. [file 41598_2023_42578_MOESM1_ESM.pdf]
